# Autologous Haematopoietic Stem Cell Transplantation in Multiple Sclerosis: a Review of Current Literature and Future Directions for Transplant Haematologists and Oncologists

**DOI:** 10.1007/s11899-019-00505-z

**Published:** 2019-03-04

**Authors:** Joyutpal Das, Basil Sharrack, John A. Snowden

**Affiliations:** 1 Sheffield Institute for Translational Neuroscience, University of Sheffield, 385a Glossop Rd, Sheffield, S10 2HQ UK; 2 Clinical Neurology, Academic Department of Neuroscience, Sheffield Teaching Hospitals NHS Foundation Trust, Sheffield, UK; 3 Blood and Marrow Transplantation, Department of Haematology, Sheffield Teaching Hospitals NHS Foundation Trust, Sheffield, UK; 4 Haemato-oncology and Stem Cell Transplantation, Department of Oncology and Metabolism, University of Sheffield, Sheffield, UK

**Keywords:** Autologous haematopoietic stem cell transplantation, Multiple sclerosis, Relapsing-remitting multiple sclerosis, Secondary progressive multiple sclerosis, Primary progressive multiple sclerosis, Aggressive multiple sclerosis

## Abstract

**Purpose of Review:**

We summarise the current development of autologous haematopoietic stem cell transplantation (AHSCT) in treating multiple sclerosis (MS) and discuss future directions for the general neurologist, transplant haematologist and oncologist.

**Recent Findings:**

AHSCT was initially performed to treat MS over 20 years ago. Over recent years, the evidence base has grown, especially in relapsing-remitting MS (RRMS), with significant improvements in safety and efficacy through better patient selection, choice of transplant technique and increase in centre experience.

**Summary:**

AHSCT is now a treatment option in very carefully selected patients with severe, treatment-resistant RRMS. However, it is important for transplant haematologists and oncologists to work closely with specialist MS neurologists in patient selection, during transplant and in long-term follow-up of patients. Data should be registered into international transplant registries and, ideally, patients should be enrolled on prospective clinical trials in order to build the evidence base and refine transplant techniques.

## Introduction

Multiple sclerosis (MS) is a chronic inflammatory disorder of the central nervous system (CNS). MS typically presents as a relapsing-remitting illness. A relapse is a discrete, self-limiting episode of neurological dysfunction, which is caused by an acute inflammatory demyelination within the CNS. Although the clinical features of a relapse may completely resolve, it usually leaves residual damage to the CNS, which may be subclinical in nature. Over time, the effect of the repeated CNS injury accumulates and leads to an irreversible and progressive neurological dysfunction, with or without superimposed relapses. Although these neuroinflammatory and neurodegenerative components run in parallel, the main pathological process of the relapsing-remitting phase is dominated by neuroinflammation, whereas in the progressive phase, it evolves into more indolent inflammation with significant progressive neurodegeneration. In many patients, the clinical transition from the relapsing-remitting (RR) to the secondary progressive (SP) phase may take 10–20 years, although in some patients, it can progress more quickly. There are no diagnostic biomarkers available for the detection of this transition which remains a retrospective assessment based largely on clinical observations. This disease model supports the existence of a ‘therapeutic window’ during which immunomodulatory interventions may prevent or delay the progressive, irreversible neurological dysfunction in patients with RRMS. In 10% of patients, the progressive neurological dysfunction starts from the onset of the illness, which is known as primary progressive (PP) MS [1, 2].

Therefore, an ideal therapeutic goal is to switch off the inflammation and halt disease progression. This is reflected in a concept that is used by neurologists termed ‘No Evidence of Disease Activity’ (NEDA); a composite endpoint of three parameters: absence of clinical relapse, disability progression and any evidence of radiological disease activity on magnetic resonance imaging (MRI) [3]. NEDA is achieved with current disease-modifying therapies (DMTs) to a variable extent.

Immunoablation and reconstitution of the immune system aiming at switching off the autoreactive, inflammatory process and restoring self-tolerance is a more intensive approach than standard DMTs in managing MS [4]. Immunoablation and autologous haematopoietic stem cell transplantation (AHSCT) was commenced as a MS therapy over two decades ago [5]. International transplant registries have progressively collected the details of several thousand patients who have received AHSCT for different forms of MS; for example, the European Society for Blood and Marrow Transplantation (EBMT) registry has now 1271 patients who have received AHSCT for MS, which forms the main indication for this treatment in the autoimmune disease database (personal communication Manuela Badoglio, EBMT Paris Office, September 2018). Recently published studies have supported the safe delivery of AHSCT whilst potentially achieving significantly higher rates of NEDA compared to DMTs in MS patients with highly active disease. In this review, we summarise the current evolution of AHSCT and discuss its future directions.

## Efficacy

A variety of conditioning regimens have been used to deliver AHSCT with variable safety and efficacy profiles, and these conditioning regimens can be divided in three categories based on intensity (Table 1) [5, 6••, 7, 8••, 9–15]. Various studies and historical registry data did not confirm any efficacy differences between these conditioning regimens [16–18].

**Table 1:** Categorisation of various conditioning regimens used for AHSCT in MS.

Intensity	Conditioning regimen examples	Ref
High	Total body irradiation, cyclophosphamide and antithymocyte globulin (ATG)	[5]
	Busulfan, cyclophosphamide and ATG	[6••]
Intermediate	Carmustine (BiCNU) 300 mg/m^2^, etoposide 800 mg/m^2^, cytarabine-arabinoside 800 mg/m^2^ and melphalan 140 mg/m^2^ (BEAM) and ATG (BEAM-ATG)	[7]
1. Myeloablative		
2. Lymphoablative	Cyclophosphamide 200 mg/Kg and rabbit ATG (Cy-ATG)	[8••]
Low	Cyclophosphamide alone	[9]
	Melphalan alone	[9]
	Fludarabine-based regimens	[10, 11]

Regardless of the conditioning regimen, AHSCT dramatically reduces annualised relapse rate and MRI disease activity in MS patients [6••, 13, 14, 19, 20••]. It may take several months to completely extinguish all MRI inflammatory disease activities, but evidence suggests that AHSCT is able to sustain radiological disease remission in the majority of treated patients [7, 14, 21, 22]. In one study, no clinical or radiological disease activity emerged during up to 13-year follow-up after immunoablation with a high-intensity conditioning regimen containing busulfan [6••]. However, breakthrough disease activity on long-term follow-up has been reported with lesser intensity conditioning regimens [7, 14, 20••, 21, 23].

The reported effect of AHSCT on disability progression varies among studies. Disability in MS is measured using the Kurtzke’s Expanded Disability Status Scale (EDSS) (Table 2) [24]. AHSCT is more effective in preventing disability progression in RRMS patients than SP MS [25–27]. AHSCT prevents EDSS deterioration in most treated patients, although in some cases, EDSS score may start to increase after several years of disease stability [14, 27–29]. Various natural history studies have suggested that disability progression is independent of relapses once a critical EDSS score is reached suggesting that neurodegeneration progresses independent of neuroinflammation [30–32]. Therefore, AHSCT may not halt disability progression in the advanced stages of the disease, despite being effective in reducing relapse rate and inducing radiological disease remission. The timing at which AHSCT is undertaken during the disease course is therefore critical to the outcome. As a result, patients with aggressive disease and shorter duration of disease are increasingly targeted as prime candidates for clinical trials.

**Table 2:** The Kurtzke’s Expanded Disability Status Scale (EDSS)

Score	Description
0	Normal neurological examination of all FS^*^
1.0	No disability but minimal signs in one FS
1.5	No disability but minimal signs in more than one FSs
2.0	Minimal disability in one FS
2.5	Minimal disability in two FSs
3.0	Fully ambulatory but moderate disability in one FS or minimal disability in three or four FSs
3.5	Fully ambulatory but moderate disability in one FS and minimal disability in one or two FS; or fully ambulatory with moderate disability in two FSs; or fully ambulatory with minimal disability in five FSs
4.0	Ambulatory without aid or rest for ≥ 500 m; self-sufficient, up and about some 12 h a day despite relatively severe disability in one FS or combination of lesser disability levels exceeding limits of previous steps
4.5	Ambulatory without aid or rest for ≥ 300 m; up and about some 12 h a day despite relatively severe disability in one FS and combination of lesser disability levels in other FSs exceeding limits of previous steps
5.0	Ambulatory without aid or rest for ≥ 200 m
5.5	Ambulatory without aid or rest for ≥ 100 m
6.0	Ambulatory with unilateral assistance ≥ 100 m with or without rest
6.5	Ambulatory with bilateral assistance ≥ 20 m without rest
7.0	Only able to ambulate ≤ 5 m with aid, essentially restricted to wheelchair; though wheels self in standard wheelchair and transfers alone; up and about in wheelchair some ss a day
7.5	Unable to take more than a few steps; restricted to wheelchair and may need aid in transferring and wheeling self
8.0	Essentially restricted to bed or chair or pushed in wheelchair, but out of bed most of day; retains many self-care functions; generally, has effective use of arms
8.5	Essentially restricted to bed much of day; has some effective use of arm(s) and retains some self-care functions
9.0	Helpless and confined to bed; can still communicate and eat
9.5	Totally helpless and confined to bed and totally dependent; unable to communicate effectively or eat/swallow
10	Death due to MS

^*^Assessment of EDSS is consist of the examination of eight functional systems (FSs)

A number of recent phase II studies, using various conditioning regimens, have reported AHSCT to be safe and efficacious over a 3–5-year follow-up period [6••, 13, 19, 20••]. HALT-MS was a phase II trial, which used BEAM-ATG, an intermediate intensity conditioning regimen, for immunoablation. In this study, 73.8% and 69.2% of patients had disease-free survival (equivalent to NEDA) at 4 and 5 years respectively [19, 20••]. Other studies from Italy, Sweden, Russia and Brazil also reported that BEAM or BEAM-like regimens were equally efficacious [7, 18, 27, 33]. An earlier single-centre, phase II trial showed that AHSCT using a different intermediate intensity conditioning regimen, 200 mg/kg cyclophosphamide and ATG (Cy-ATG), was able to achieve NEDA in 62% of cases at 3 years [23]. A large case series of 145 patients (118 patients with RRMS and 27 patients with SPMS) treated in one centre showed that similar efficacy figures with 80% and 68% of patients had NEDA at 2 and 4 years respectively [8••]. A Canadian multicentre, phase II trial showed that AHSCT using a high-intensity conditioning regimen containing busulfan, cyclophosphamide and ATG was able to induce NEDA in 69.6% of patients with aggressive disease at 3 years, although the use of busulfan in the containing regimen was associated with veno-occlusive disease of the liver in two patients, one of whom died [6••].

Considering the heterogeneity of the conditioning regimens and MS phenotypes, an attempt has been made to integrate the outcomes of the various studies using meta-analysis which reported that cumulative NEDA was achieved in 83% (range 70–92%) of patients at 2 years and 67% (range 59–70%) of patients at 5 years [34]. However, other data suggested that yearly NEDA could be sustained over 10 years and beyond [6••, 21, 27]. Although these studies lack control groups, AHSCT remains highly efficacious and may be superior to high-efficacy DMTs, such as Alemtuzumab, Natalizumab, Ocrelizumab and Cladribine which achieve yearly NEDA in 27–62% of treated patients at 2–5 years (Table 3) [35–39].

**Table 3:** Mechanism of action and the rate of yearly NEDA with high-efficacy DMTs

Drug	Mechanism of action	Rate of NEDA	Ref
Alemtuzumab	A humanised monoclonal antibody selectively targeting CD52 highly expressed on T and B lymphocytes	58.2^a^–62.4^b^ % at 5 years	[35, 36]
Natalizumab	α_4_ integrin antagonist, a selective adhesion molecule inhibitor	27^c^–40^d^ % at 2 years	[37]
Ocrelizumab	A humanised anti-CD20 antibody	48% at 96 weeks	[38]
Cladribine	A synthetic deoxyadenosine analogue which induces a preferential and sustained reduction in numbers of circulating peripheral T and B lymphocytes	47^e^ % at 96 weeks	[39]

These are yearly NEDA rate and likely to be significantly higher than cumulative NEDA rate over 5 years

^a^Patients were treated with DMT prior to participating in the trail

^b^Patients were treatment naïve before receiving Alemtuzumab

^c^Patients had non-highly active disease, which was defined as fewer than two relapses or no gadolinium-enhancing lesions at study entry

^d^Patients had highly active disease, which was defined as at least two relapses in the year before study entry and at least one gadolinium-enhancing lesion at study entry

^e^Patients were treated with 3.5 or 5.25 mg/kg of Cladribine

These encouraging developments need to be considered with the caveat that there is a lack of fully published randomised controlled trials (RCTs) directly comparing AHSCT with current standard DMTs. The first such trial was the EBMT ‘ASTIMS’ trial which was a multicentre, phase II RCT of AHSCT against mitoxantrone, an agent which is now rarely used in the era of biological MS therapy [40•]. In this study, AHSCT was more effective in reducing MRI disease activity and annualised relapse rate than mitoxantrone over 4 years. The study cohort was small, and the majority of patients had progressive disease, and it was therefore not surprising that there was no statistically significant difference on disability progression.

The ‘MIST’ trial (clinicaltrials.gov identifier: NCT00273364) is the only multicentre, randomised phase III trial, which is comparing the efficacy of AHSCT with the best medical management in patients with RRMS [41•]. In the DMT control arm, patients were managed with a range of DMTs, but notably not with the more recently approved Alemtuzumab or Ocrelizumab. The primary endpoint was treatment failure, which was defined as an increase of at least 1.0 point of EDSS sustained for 6 months. With a median follow-up of 2 years (range 1–5 years), this was observed in 67% of patients in the DMT arm compared to 6% of patients in the AHSCT arm. The full report is expected in 2021. However, the question remains as whether AHSCT is more effective than high-efficacy DMTs, such as Alemtuzumab or Ocrelizumab.

A number of studies have reported that the reduction of EDSS score following AHSCT indicated an improvement of disability [8••, 13, 20••, 27]. This improvement often lasts for many years, suggesting that if the CNS inflammation is adequately suppressed for a sufficient period of time, functional recovery may occur, which is mediated not only by the immediate benefits from the arrest of inflammation, but also through repair and regenerative mechanisms in the longer term. Once the reserve capacity of a neural network is completely exhausted, it may lose its ability to make spontaneous recovery and to improve its function [42]. Modest improvement of EDSS has also been reported with some high-efficacy DMTs, such as Alemtuzumab [35, 43]. Although more clinical and scientific evidence is required, the induction of profound disease remission early in the disease course with AHSCT followed by partial or complete reversal of disability contradicts the long-standing notion that disability related to disease progression in MS is irreversible.

## Safety and Tolerability

Early complications of AHSCT include cytopenia, transient alopecia, fever, engraftment syndrome, mucositis, infection and other toxicities common to all autologous transplant procedures [8••, 18]. Transient neurological worsening may also be a relatively unique feature to this group of patients. In particular, fever due to ATG and/or infection may exacerbate neurological symptoms such as pain, spasticity, weakness and fatigue. Febrile neutropenia or sepsis requires urgent assessment, investigations and treatment with antimicrobial therapies, but also consideration of whether it could be related to the effect of ATG, which may persist significantly beyond the conditioning phase. Corticosteroids and paracetamol should be considered to prevent prolonged pyrexia in the absence of infection. Peri-transplant-sustained pyrexia regardless of the presence or absence of infection has been associated with poor long-term neurological recovery in one study [8••]. Therefore, prompt action should be considered to prevent prolonged pyrexia from whatever the cause might be.

Reactivation of varicella-zoster virus is a common late complication, perhaps due to the more intense immunosuppression associated with ATG used in the conditioning regimen in this setting. Secondary autoimmune conditions following AHSCT are encountered with a greater frequency compared to the malignant disease setting. This includes autoimmune thyroiditis, immune thrombocytopenic purpura (ITP), rheumatoid arthritis, Crohn’s disease and acquired anti-factor VIII inhibitor [6••, 16, 25, 28]. However, the rate of secondary autoimmune conditions with AHSCT appears to be significantly less than treatment with Alemtuzumab, where almost half of the patients develop a secondary autoimmune condition [44].

Other late complications include the development of late malignancy and infertility, which probably occur less frequently compared to the malignant disease setting. Post-transplant lymphoproliferative disease, glioblastoma multiforme, breast cancer, squamous cell carcinoma, prostate cancer and cervical cancer have been reported so far [16, 22, 25, 28]. AHSCT causes temporary or permanent ovarian and testicular failure, and patients should be counselled thoroughly before AHSCT. Even so, some females treated with AHSCT for autoimmune diseases naturally conceived pregnancies and gave birth to healthy babies [45]. In those circumstances, menses recommenced around 3–4 months after AHSCT [45]. Similarly, men can also father healthy babies following AHSCT. Fertility conservation procedures, such as cryopreservation of sperm, egg or embryo should be considered for all patients undergoing AHSCT. In addition, hormone replacement therapies should be offered where appropriate.

However, the main concern limiting the use of AHSCT for MS has been the risk of treatment-related mortality (TRM). The analysis of the data from EBMT registry revealed a dramatic decline in TRM over the last two decades despite the increased use of AHSCT for MS. The TRM rates were 7.3% between 1995 and 2000, 1.3% between 2001 and 2007 and 0.7% between 2008 and 2016 [46]. It is also necessary to recognise that high-efficacy DMTs have various short- and long-term toxicities, including progressive multifocal leukoencephalopathy and other serious infective complications, which are also associated with significant morbidity and mortality risks [35, 47, 48]. In our centre, even though we have not had any TRM in MS patients, our practice is to state to all patients that this procedure is associated with an approximately 1% risk of TRM, which is also in keeping with TRM rate for AHSCT across common haemato-oncological indications, such as myeloma, lymphoma and solid tumours. It is prudent to inform patients adequately.

‘Intermediate’ conditioning regimens have been the most widely used conditioning regimens in the EBMT registry. The international data registries have yet to show any significant advantage between myeloablative and non-myeloablative intermediate conditioning regimens, and, in addition, there is a lack of data about their relative secondary complication rates [12, 16]. In the EBMT Autoimmune Diseases Working Party, registry studies are in progress to evaluate outcomes and safety, including TRM and long-term complication rates (‘late effects’) in order to help define the best transplant technique in MS.

## Reasons for the Improvement of Efficacy and Safety

There are three key factors behind the recent improvement in safety and efficacy of AHSCT: (1) patient selection, (2) choice of transplant regimen and (3) centre experience.

### Patient Selection

Recently, it has become evident that AHSCT is more efficacious in patients with RRMS than SPMS or PPMS [25–27]. EBMT registry also reflects these findings, as the proportion of RRMS patients receiving AHSCT has increased over the years compared to progressive forms of MS [17]. As an intensive anti-inflammatory treatment, it is far more logical for AHSCT to be used as an induction therapy for treatment-refractory aggressive disease rather than a salvage therapy for progressive forms of MS. The natural history of MS is that it takes 20 years for patients to lose their ability to ambulate without assistance and it takes another few years to become wheelchair bound [30]. Unfortunately, about 4–14% of patients who have aggressive disease experience an accelerated disease course [49, 50]. In this group of patients, the disease progresses three to four times faster. Various terminologies have been used to describe this phenotype including ‘aggressive’ MS, ‘malignant’ MS, ‘highly active’ MS and ‘fulminant’ MS. The ‘therapeutic window’ in a patient with ‘aggressive’ MS is significantly shorter due to the rapidly progressive nature of the disease and, in reality, it is often a retrospective diagnosis. If these patients are not treated, they will become severely disabled within a few years. Early accrual of disability is the hallmark of this form of MS. It does not respond to first-line DMTs such as Beta interferon or Glatiramer acetate and requires early introduction of Alemtuzumab, Natalizumab, Cladribine or Cyclophosphamide, but a proportion of patients will not respond even to these drugs [50, 51]. Patients should be closely monitored for treatment failure. AHSCT should be considered if there is any evidence of breakthrough disease activity, preferably earlier in the disease course before irreversible disability develops.

Several other demographic- and disease-related characteristics also influence the treatment outcome. Younger patients, shorter disease duration, lower EDSS scores, active inflammatory disease, and absence of other co-morbidity have been associated with favourable outcomes [8••, 26–28, 52, 53]. These are interlinked; the shorter disease duration means that patients are likely to be in their relapsing-remitting phase of illness and thereby also likely to have lower EDSS. In the absence of any other significant co-morbidity, younger patients would tolerate the toxic chemotherapy regimen better than older patients with multiple co-morbidities. As AHSCT results in rapid cessation of all inflammatory disease activity, it is not surprising that MS patients with evidence of active inflammation do better with AHSCT than patients with indolent inflammation. Several reports found that the presence of gadolinium enhancement at pre-AHSCT MRI was associated with favourable outcomes, even in patients with the progressive forms of MS [7, 27]. Gadolinium enhancement indicates a breakdown of the blood-brain-barrier, which may facilitate the CNS penetration of the conditioning regimen and enhance the elimination of autoreactive immune cells. In 2012, the EBMT published guidelines and recommendations about the use of AHSCT for MS. These guidelines suggest that AHSCT may be offered to those patients who have RR disease and able to ambulate independently, but experience two clinical relapses with MRI evidence of concurrent disease activity in the previous year despite the use of standard DMTs. Patients unable to ambulate independently due to rapid accumulation of disability from aggressive disease, or occasionally patients with progressive disease with clear evidence of significant clinical and MRI disease activities may be considered for AHSCT even though the benefit in patients with the progressive forms of MS is more limited [12]. In addition to the guidelines, current consensus also advocates its use in patients who are below 45 years of age and have disease duration less than 10 years [54].

### Choice of Conditioning Regimen

The myeloablative ‘intermediate’ intensity BEAM-ATG conditioning regimen which was derived from lymphoma treatment regimens has historically been the most popular conditioning regimen in Europe and also widely used in North and South America [9, 16]. This is used for immunoablation in patients with MS rather than other autoimmune conditions treated with AHSCT. Since the publication of the 2012 EBMT guidelines, there has been an increase in use of the non-myeloablative, intermediate intensity Cy-ATG conditioning regimen, which was originally derived from aplastic anaemia treatment regimens [55]. It is the generic regimen that is used for immunoablation in all autoimmune conditions, including MS treated with AHSCT. One study attempted to deescalate the dose of cyclophosphamide in this intermediate conditioning regimen from 200 to 120 mg/kg, but this regimen was unable to suppress the MRI disease activity [15]. Total body irradiation is discouraged due to greater short- and long-term risks, including infections, secondary malignancies, TRM and EDSS progression possibly due to neurotoxicities, and is now rarely used, if at all, in MS. Likewise, the busulfan containing high-intensity conditioning regimen has fallen out of use in Europe since the early 2000s [9, 52].

A higher-intensity conditioning regimen is likely to have a higher efficacy, but this superior efficacy may be offset by its higher rate of toxicities. Therefore, in clinical practice, a balance between the intensity and the safety of the treatment has to be achieved. Two intermediate regimens: the myeloablative BEAM-ATG and the non-myeloablative Cy-ATG regimens have shown to induced high rates of sustained NEDA and no TRM in several recent studies demonstrating a good balance between efficacy and safety [7, 8••, 20••]. At present, there is no clear comparative data as to the relative efficacy and safety of these two most commonly used intermediate conditioning regimens [16–18] and therefore, EBMT guidelines advocate using either of these two regimens for MS [12]. The question of relative superiority between these two intermediate treatment regimens may be resolved through registry analysis, but ideally RCTs should be performed.

### Centre Experience

Interrogations of the data from the EBMT and the Center for International Blood and Marrow Transplant Research (CIBMTR) registries revealed that the TRM was associated with the experience of the treatment centre [9, 16]. It is recommended that AHSCT for autoimmune conditions including MS should be performed by Joint Accreditation Committee ISCT-Europe & EBMT (JACIE)-approved (or equivalent) centres. In a transplant centre, the care service is usually tailored for haemato-oncology patients, as they form the majority of the patient population. There needs to be a consideration for MS specific supportive care measures, including multi-disciplinary teams, which are likely to develop with the increase in centre experience. Neurologists must have experience in selecting appropriate candidates for AHSCT, whereas haematologists need awareness, knowledge and experience of managing various early treatment-related complications, such as sustained fever. Although patients typical do not require any further immunosuppressive therapy, they require long-term monitoring for various late transplant-related complications as well as for symptoms and signs of breakthrough disease activity. MRI brain and/or spine at regular intervals are required post-transplant, as indolent inflammation may not be clinically apparent. There are many other additional considerations. For example, many MS patients treated with AHSCT are in the childbearing age group. Temporary ovarian/testicular failure and infertility following AHSCT are known risks and therefore require counselling, and necessary care.

## Expert Opinion and Future Prospects

Currently, there are no RCTs comparing the AHSCT with high-efficacy DMTs. In the UK, a multicentre, randomised, phase III trial (STAR-MS) will start recruiting patients from 2019 and randomise them to either AHSCT or Alemtuzumab. Other similar international phase II/III RCTs are also attempting to address similar key issues, such as RAM-MS and BEAT-MS. RAM-MS is an international, multicentre, randomised trial comparing AHSCT with Alemtuzumab whereas BEAT-MS is a follow-up phase III trial from the HALT-MS phase II trial. In addition, there is a case for future clinical trials with novel conditioning regimens that can deliver disease-specific or targeted immunoablation with an aim to further reduce toxicities whilst maintaining the efficacy compared to the current regimens.

Health economic evaluation of AHSCT treatment will be central to financing any publicly or privately funded service. MS does not only cause a massive burden of costs from long-term treatment with DMTs, but also reduces long-term quality of life and leads to unemployment, progressive disability and eventually dependency, which costs substantially to the individual and the health care service. Although AHSCT is not cheap, it is a one-off treatment and the therapeutic benefits are likely to sustain for many years suggesting that there may be major health economic benefits. Hence, health economic studies evaluating the delivery of AHSCT versus DMTs are needed in the future across a range of health care services.

Another future challenge in the field of MS is to whether any benefit can be derived from AHSCT for the progressive forms of the disease. In the last two decades, a large number of patients with progressive disease have been treated with AHSCT [9, 16]. Some studies offer support that AHSCT could reduce the relapse rate and progression of disability in the progressive forms of MS, but it is difficult to interpret these studies due to the lack of control groups [14, 28, 55]. Therefore, this limited therapeutic benefit must be weighed against the potential higher rate of TRM associated with advance disability in SPMS on an individual case basis before offering AHSCT. Further, RCTs are required to assess the therapeutic benefit of AHSCT in SPMS. Based on the earlier registry studies, AHSCT does not appear to be effective in PPMS and therefore was not recommended in EBMT guidelines [12, 52]. However, recent studies with DMTs including Beta interferon, Ocrelizumab and Rituximab have suggested that primary progressive MS may respond to immunomodulation highlighting that inflammation plays an important part in progressive phase of the disease [56–58]. Further studies are required to explore its potential as a therapy for PPMS.

There are two other avenues in cellular therapy that require further exploration. For progressive forms of MS, multipotent mesenchymal stromal cells may have some promise as an immunomodulator and may also possess the ability to promote remyelination [59, 60]. Genetic manipulation of haematopoietic stem cells ex-vivo to develop self-tolerance against myelin epitopes has also been through early stages of investigation [61].

## Conclusions

AHSCT is now evolving as a highly efficacious and relatively safe therapeutic option for treatment-refractory RRMS patients. Whether AHSCT offers a potential cure remains unknown, and in addition to sustained efficacy, long-term safety considerations remain of paramount importance. Clinical trials and long-term registry data are required to ascertain the long-term safety and efficacy of AHSCT over DMTs and to optimise transplant techniques. Health economic factors, such as long-term quality of life, employment and costs associated with treatment and supportive care for AHSCT relative to DMTs have to be studied for further improvement of patient care and service. The safety of this treatment has a direct positive correlation with the experience of the treatment centre. In addition, a multi-disciplinary team that consists of transplant specialists, MS neurologists, other supporting clinicians (such as fertility services) and various allied health care professionals is essential for the best clinical practice in this field.
